# A Case of Hyalinizing Clear Cell Carcinoma, So-Called Clear Cell Carcinoma, Not Otherwise Specified, of the Minor Salivary Glands of the Buccal Mucosa

**DOI:** 10.1155/2015/471693

**Published:** 2015-10-27

**Authors:** Takahiro Yamanishi, Kiwako Kutsuma, Keisuke Masuyama

**Affiliations:** Department of Otolaryngology-Head and Neck Surgery, Faculty of Medicine, University of Yamanashi, 1110 Shimokato, Chuo, Yamanashi 409-3898, Japan

## Abstract

Hyalinizing clear cell carcinoma (HCCC), so-called clear cell carcinoma, not otherwise specified (CCC (NOS)), of the salivary glands is a rare and low-grade malignant tumor. We report a case of HCCC so-called CCC (NOS) (referred to as HCCC) of the minor salivary gland of the buccal mucosa. A 52-year-old woman had presented with a gradually growing and indolent mass in the right buccal mucosa for about two years. The first biopsy histopathologically suggested the possibility of malignancy derived from the minor salivary glands. A month later, she visited our hospital. The tumor measured approximately 1.5 cm in diameter and was elastic hard, smooth, and well movable. Image examinations demonstrated internal homogeneity of the lesion, which had a smooth margin, in the right buccal mucosa. Complete tumor resection followed by covering with a polyglycolic acid sheet and fibrin glue spray was performed without surgical flap reconstruction. Histopathological findings revealed proliferating tumor cells with clear cytoplasm surrounded by hyalinizing stroma in the submucosal minor salivary glands. Immunohistochemical stains revealed these tumor cells to be positive for epithelial cell markers but negative for myoepithelial ones. These findings confirmed the diagnosis of HCCC. Good wound healing and no evidence of local recurrence and metastasis have been shown since surgery.

## 1. Introduction

Hyalinizing clear cell carcinoma (HCCC), so-called CCC, not otherwise specified (NOS), is an epithelial malignant tumor that occurs in the salivary glands, kidney, lung, thyroid, parathyroid, and female reproductive organs. HCCC in the salivary glands was first reported in 1994; it was described that HCCC occurred predominantly in the intraoral minor salivary glands, more commonly in middle-aged women [1]. It is a rare tumor that represents less than 1% of all malignant tumors in the salivary glands. The most common locations are the palate and tongue, which account for almost 50%, while it occurs less frequently at the buccal mucosa. As mentioned above, this condition occurs predominantly in the minor salivary glands, accounting for only about 10% of all salivary gland tumors [2, 3]. It is a low-grade malignant tumor classified as one subtype of salivary gland tumor in the revised WHO classification in 2005 [3]. Because HCCC is a low-grade malignancy, slow and indolent growth is one of the most important features. The latest classification of salivary gland tumors is the first to use the term “clear cell carcinoma, not otherwise specified” and considers it a diagnosis of exclusion [2, 3].

Histopathologically, the tissue of HCCC is composed of proliferating epithelial cells with clear cytoplasm, organized in trabeculae, cords, or solid nests surrounded by hyalinizing fibrocollagenous stroma [1, 4]. However, the differential diagnosis can be difficult because the microscopic features of HCCC frequently overlap with those of other salivary gland tumors and metastatic renal cell carcinoma. Immunohistochemical staining is effective and can differentiate it from other tumors. HCCC cells are positive for epithelial cell markers such as cytokeratin and negative for S-100 protein, mucicarmine, and myoepithelial cell markers such as SMA, MSA, myosin, and calponin [1, 4].

Complete tumor resection is essential as the treatment, and surgical flap reconstruction is needed in some cases. Recurrence or distant metastasis after complete resection is uncommonly documented. Here, we describe a case of rare HCCC of the minor salivary glands arising at the buccal mucosa, with a review of the literature.

## 2. Case Report

The patient was a 52-year-old woman presenting with a gradually growing and indolent mass at the right buccal submucosa. Her past medical history and familial history were unremarkable. From about two years previously, she had noticed the mass, but it had been left untreated because of being painless and not showing rapid enlargement. However, it also showed no tendency to improve, and then its swelling gradually worsened.

**Figure 1:**
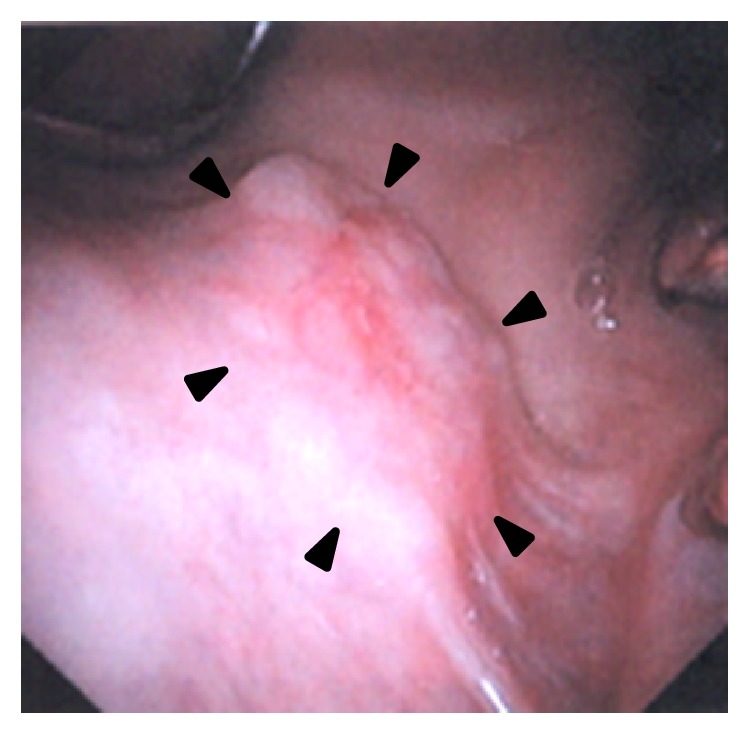
Local findings. A smooth, elastic hard, and exophytic mass was detected in the right buccal mucosa. Its surface was not erosive or ulcerated. Two tooth crowns can be seen on the right side of this figure.

**Figure 2:**
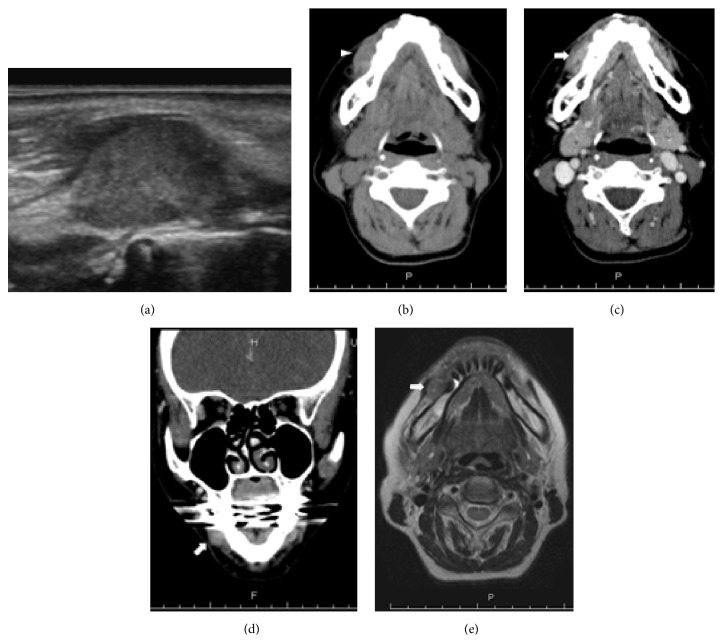
Findings of image examinations of the neck. (a) Echo showed a homogeneous hypo- to isoechoic lesion with a smooth margin. (b) Plain CT, axial view. Homogeneous low-density lesion (arrowhead). (c) Contrast-enhanced CT, axial view. (d) Contrast-enhanced CT, coronal view. Faintly enhanced lesions (arrows) were noted. (e) MRI, T2-weighted image, axial view. MRI showed a homogeneous low- to isointense lesion with partially high intensity (arrows).

At the time of her consultation at a primary care clinic for otorhinolaryngology, a cartilage-like elastic hard mass was palpable in the right buccal mucosa, so incisional biopsy was performed. The histopathological diagnosis suggested the inclusion of malignant tumor derived from the minor salivary glands, such as adenoid cystic carcinoma or mucoepidermoid carcinoma, in the differential diagnosis. A month later, she first visited our department. On physical examination, a smooth-surfaced, nontender, elastic hard, well-movable, and approximately 1.5 cm exophytic mass was detected in the right buccal mucosa. The overlying mucosal surface had no erosion or ulceration (Figure 1). There were no abnormal findings in the ear, nose, throat, head, and neck, as well as no cervical lymphadenopathy, except for the buccal mass. Neck echo showed a hypo- to isoechoic lesion with a smooth margin measuring 12.5 mm × 9 mm (Figure 2(a)). CT and MRI showed an internally homogeneous and enhanced lesion measuring 1.3 cm in the greatest dimension in the right buccal mucosa; in addition, no evidence of cervical lymph node metastasis or distant metastasis was found (Figures 2(b) and 2(c)). No significant abnormalities were also noted in the laboratory examinations, including for tumor markers.

**Figure 3:**
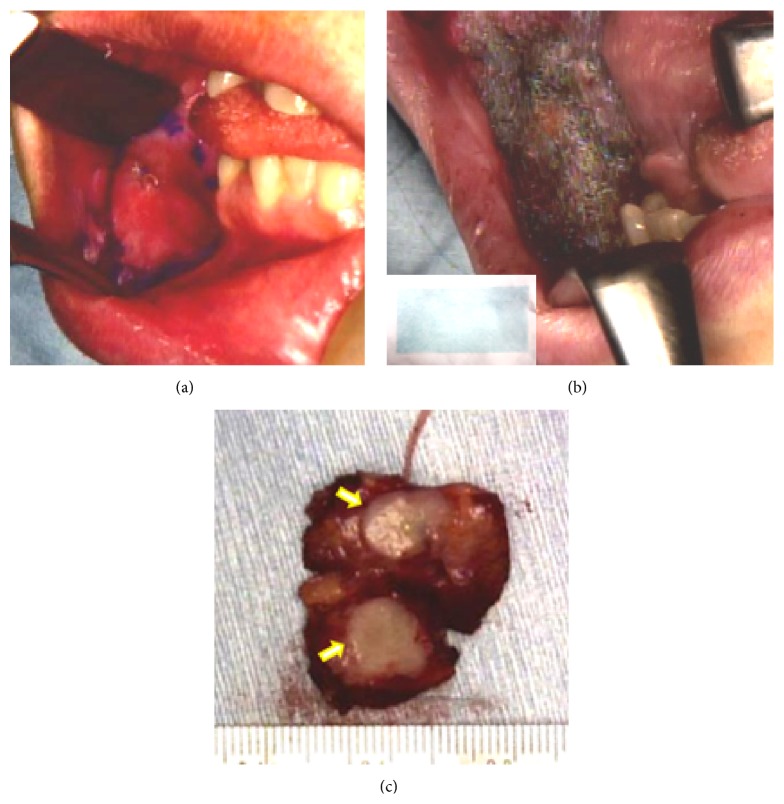
Operative findings. (a) The incision line (purple circle on the right buccal mucosa) was designed with a safety margin of about 1 cm around the tumor. (b) The wound was covered with a graft using a polyglycolide acid sheet (PGA sheet, NEOVEIL; Gunze Ltd., Kyoto, Japan) and fibrin glue spray. The inserted figure at the lower left is from before use of the PGA sheet. (c) Cut surface of the resected tumor. Its section revealed a solid yellowish white cut surface with a smooth margin, without adhesion of the surrounding tissue.

Complete surgical resection of the tumor was recommended and performed. This resection was carried out with a safety margin of about 1 cm around the tumor (Figure 3(a)). The margin of depth was set as the buccal subcutaneous connective tissue. Negative resection margins were confirmed by intraoperative frozen section analysis. After complete tumor resection, the open wound was covered with the graft using a polyglycolide acid sheet (PGA sheet, NEOVEIL; Gunze Ltd., Kyoto, Japan) and fibrin glue spray (Figure 3(b)). Surgical flap reconstruction and neck dissection were not performed. Postoperative tube feeding was started for wound healing, followed by ingestion on postoperative day 6.

Grossly, the tumor was circumscribed and showed a solid yellowish white cut surface. It measured 1.1 cm in its greatest dimension, without adhesion of the surrounding tissue (Figure 3(c)).

**Figure 4:**
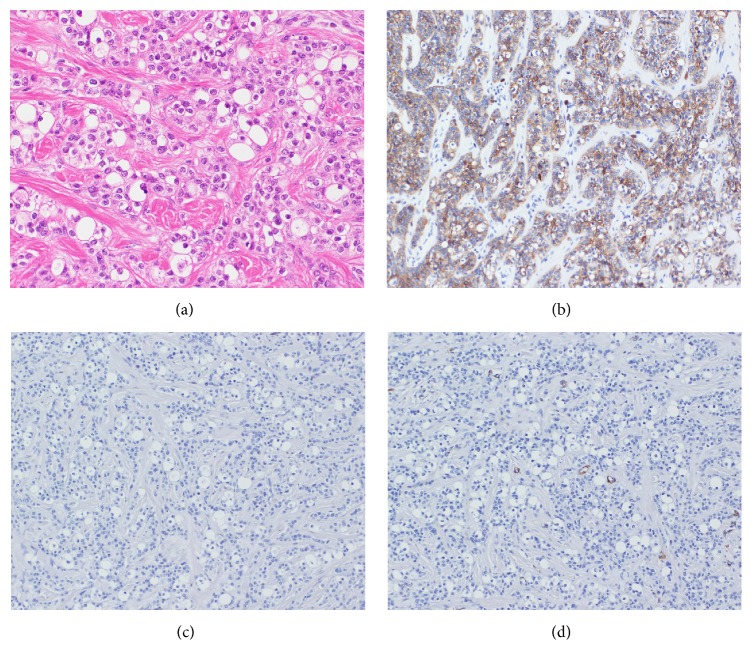
Histopathological and immunohistochemical findings ((a) HE stain ×200, (b)–(d) immunohistochemical stain ×200; (b) pancytokeratin (AE1/AE3); (c) S-100; (d) SMA). Proliferation of tumor cells with clear cytoplasm organized in trabeculae, cords, or solid nests surrounded by hyalinizing stroma. The nuclei of the cells were small and round in shape. Tumor cells were stained intensely with pancytokeratin, but not S-100 and SMA.

Histopathologically, the invasive proliferation of tumor cells with clear cytoplasm was arranged in trabeculae, cords, or irregular solid nests surrounded by hyalinizing stroma in the submucosal minor salivary glands (Figure 4(a)). The tumor cells demonstrated small nuclei, mild to moderate atypia, and little mitosis. Mucus-producing cells and acinar cells were also rarely observed. Immunohistochemically, the tumor cells were positive for cytokeratin AE1/AE3 (Figure 4(b)) and p63, consistent with epithelial cells, but negative for vimentin, S-100, and SMA (Figures 4(c) and 4(d)). The CRT1/3-MAML2 fusion gene, which is specific for mucoepidermoid carcinoma, was not detected by RT-PCR (data not shown). These microscopic examinations of the final specimen confirmed the diagnosis of HCCC, so-called CCC (NOS), with negative resection margins.

**Figure 5:**
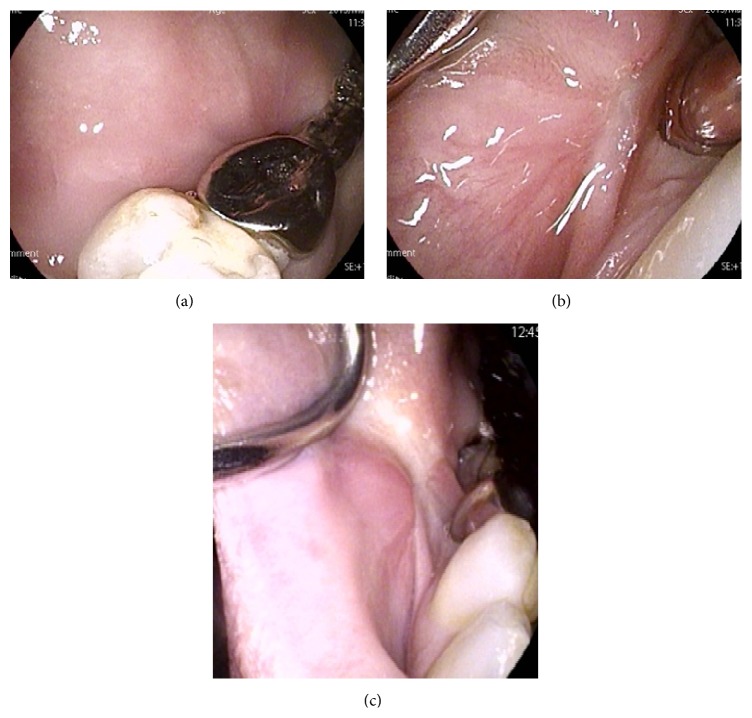
Postoperative local findings. Stable epithelialization of the wound by renewed buccal mucosa was observed. Good wound healing was also noted ((a) and (b) three months later, (c) five months later).

The patient's postoperative course has been uneventful. No significant complications such as facial palsy or scar and stable epithelialization by renewal buccal mucosa have been observed (Figure 5). She has not undergone postoperative adjuvant therapy. For a postoperative follow-up period of five months, good wound healing and no evidence of local recurrence and cervical lymph node or distant metastasis have been exhibited. We have continued with a regular follow-up.

## 3. Discussion

HCCC, so-called CCC (NOS), of the salivary glands is a rare and low-grade malignant tumor. HCCC of the salivary glands was first reported in 1994 [1]. It is involved in less than 1% of all malignant tumors in the salivary glands. A total of 90% of such cases occur predominantly in the minor salivary glands, which account for only about 10% of all salivary gland tumors. The most commonly affected sites are the tongue and palate, which constitute almost 50% of the total. The rarity of HCCC in the major salivary glands is one of its most important features. Our case was derived from the minor salivary glands of the buccal mucosa, although the rarity of HCCC of the buccal mucosa was also previously reported [2, 5–7]. It was reported that more than 60% of HCCC occurred in middle-aged women over 50 years old, and the local tumor size of more than 55% of them measured less than 2 cm in the longest dimension [5]. Our case was in the majority in both regards. It was an epithelial malignant tumor that was first defined as CCC (NOS) and classified as one subtype of salivary gland tumor in the revised WHO classification in 2005. CCC (NOS) was reported to include HCCC, clear cell adenocarcinoma, and glycogen-rich adenocarcinoma in the old classification [2, 3]. The features of histopathological HCCC are as follows: (1) the invasive proliferation of tumor cells organized in trabeculae, cords, or irregular solid nests surrounded by hyalinizing stroma in the perilesional tissue of salivary glands and (2) tumor cells with clear cytoplasm and circular or polygonal nuclei [6–9].

However, HCCC or CCC (NOS) has been considered a diagnosis of exclusion [2, 3]. Clear cells in salivary gland tumors are found in various tumor tissues, such as epithelial myoepithelial carcinoma, mucoepidermoid carcinoma, acinar cell carcinoma, sebaceous carcinoma, and metastases from renal cell carcinoma [7, 9]. Therefore, it is not possible to distinguish the other tumors by only the existence of clear cells. Each tumor has its own characteristic tissue structure, so both microscopic and immunohistochemical examinations are essential for differentiating tissue types of tumors containing clear cells [10]. HCCC is further supported by the expression of cytokeratin and p63 and the lack of expression of S100 and SMA [9]. On the other hand, mucoepidermoid carcinoma is characterized by a mixture of epidermoid and columnar mucus-producing cells. The stroma is rarely hyalinized. Both morphology and a positive reaction for mucicarmine strongly point to a diagnosis of mucoepidermoid carcinoma [11]. The clear cells of epithelial myoepithelial carcinoma are positive for S-100 protein, MSA, and SMA [6]. Acinic cell carcinoma has an acinar structure and contains fewer clear cells than HCCC. Metastatic clear cell renal cell carcinoma shares many histologic and immunohistochemical features with HCCC. The presence of prominent sinusoids, hemorrhage, and hemosiderin deposition, as well as the coexpression of cytokeratin and vimentin, strongly suggests a diagnosis of clear cell renal cell carcinoma. Renal evaluation is required in some cases [7, 9, 11]. It is complicated but important to differentiate HCCC from other tumors with clear cells since their treatment and clinical outcome differ [8].

In our case, the CRT1/3-MAML2 fusion gene specific for mucoepidermoid carcinoma was not detected. The rearrangement of Ewing sarcoma breakpoint region 1 (EWSR1) and EWSR-ATF fusion have been described in salivary gland HCCC [9]. It has been reported that 87% of HCCC demonstrated the* EWSR1* rearrangement [12]. Since EWSR-ATF fusion specific for HCCC can be evaluated by RT-PCR, it will be possible to use EWSR1-ATF fusion and EWSR1 rearrangement in HCCC as hallmark markers that may assist in differentiating it from other salivary neoplasms.

Complete local resection is considered the recommended treatment of HCCC. Although a report has shown a significant therapeutic effect by CDDP and 5-FU combination chemotherapy [2], the efficacy of radiation therapy and chemotherapy has been controversial. It has been asserted that their therapy should be chosen only for cases with postoperative recurrence or positive tumor resection margins. However, especially in cases of buccal mucosa origin, because of the close proximity to the face of the tumor in terms of the surgical resection margins, surgical reconstruction may be required after excision to prevent facial wounds and scar formation. As surgical flap reconstruction is invasive, original motility and flexibility of buccal mucosa and facial muscle may be impaired.

We performed tumor resection followed by tissue defect and wound covering using a PGA sheet and fibrin glue spray. Surgical flap reconstruction was not required. PGA sheets have already been widely used in thoracic surgery and neurospinal surgery [13, 14] and are also frequently used for tissue defect and wound covering after partial glossectomy for lingual malignant tumor [15]. They are very effective to cover wounds and repair tissue defects. This method provides great benefits in terms of good wound healing and mucosal epithelialization, minimally invasive surgery, and esthetic outcome. On the other hand, the disadvantages are that the patients cannot ingest for several days in order to rest the postoperative wound and are forced to undergo nasogastric tube feeding. At our department, they are generally instructed to restart drinking on postoperative day 5 and ingestion of food on day 6 or 7.

HCCC is a low-grade tumor, the slow and indolent growth of which is one of its most important features [4]. As recurrence, cervical lymph node metastasis and distant metastasis of HCCC after complete surgical resection are uncommonly documented, and the majority of patients have a favorable prognosis [4, 6–8, 10]. However, 3 cases of cervical lymph node metastasis and 2 cases of distant metastasis, such as to the lung, after resection were previously reported [7, 8]. These findings imply that HCCC is less indolent than was previously believed, so we should not be optimistic and appropriate care should be taken. Prophylactic neck dissection performed at the time of primary surgical resection is now controversial. If neck dissection is not performed even in N0 cases, careful and regular follow-up with attention to the cervical lymph nodes and distant metastasis would be essential in the management of HCCC [4, 7, 16].

## 4. Conclusion

HCCC of the salivary glands is a rare and low-grade malignant tumor. Histopathological and immunohistochemical evaluations are mandatory for its definitive diagnosis. In particular, its immunohistochemical pattern, expression of cytokeratin and p63, and lack of S100 and SMA are further suggested. Complete local resection is essential. In our case, the open wound was covered with a graft using a PGA sheet and fibrin glue spray after tumor resection. Good wound healing, mucosal epithelialization, and no facial scar were noted. No evidence of local recurrence or cervical lymph node or distant metastasis has been observed. Although it is a low-grade malignant tumor, regular and careful follow-up is important.
